# Graduate Study on the Continent.—Germany

**Published:** 1908-05-16

**Authors:** 


### HOSPITAL ADMINISTRATION.

### CONSTRUCTION AND ECONOMICS.

### GRADUATE STUDY ON THE CONTINENT.—GERMANY.

III.—GRADUATE STUDY ASSOCIATIONS OF BERLIN.

A LIST of graduate study associations who cater for the qualified practitioner may not be without use to those who intend to make Berlin their centre. First to be mentioned—not because it is the largest, but because it is an excellent society which has done and is doing work that demands wider recognition and appreciation than it has hitherto received—is the Anglo-American Medical Association, which was founded in 1903 by Dr. James H. Honan, of Berlin, who invited the American and English practitioners engaged in post-graduate study in this city to a meeting at his house. The society has its office and an excellent reading-room, where the more important German and foreign medical journals and the latest monographs and text-books may be seen, at Rothackers Buchhandlung, Friedrichstrasse 105 b. Here the representative of the Association is at the disposal of visitors every morning from 11 to 12, to assist them personally in house or room hunting, and to arrange courses, etc., for them. Every Saturday evening at half-past seven the Association meets in room 5 at the Heidelberg Restaurant, in the Central Hotel Building, near the Friedrichstrasse Station, and visitors are cordially welcomed. On the lecture programme appear such well-known names as those of Professors Cohnheim, Dührssen, Knorr, Miller, Krause, Baginsky, Schleich, Bumm, Senator, Meyer, and Goldsmidt. Every week at these meetings an interesting post-graduate clinic is given by some well-known medical teacher, the lectures being purely practical and such as would interest men who are interested in some special subject. After the lecture the Secretary gives out notices regarding new courses, new members are formally enrolled, paying a small fee (5 marks), and any other business on the agenda is proceeded with. As an example of the activity of the Association, we cannot do better than give a list of the various courses which are given at present under its auspices :—

*Internal Medicine.*—Professor Michaelis, daily, 4 weeks, 40 marks. Professor Brandenburg, 3 hours weekly, 4 weeks, 40 marks. Professor Strauss, twice weekly, 4 weeks, 40 marks. Drs. Klemperer, Jacobson and Mosse, each a 4 weeks' clinical course, 40 marks. Professor Lazarus, special course. Dr. Steyrer, special course.

*Stomach and Intestines.*—Drs. Glücksmann, Elsner, and Ury, each a 4 weeks' course, fee 50 marks. Professor Albu and Cohnheim, each a 4 weeks' special course, fee 50 marks.

*Surgery.*—Oberarzt Dr. Braun, 4 weeks, 2 hours three times weekly, special operative surgery on the cadaver, fee 70 marks. Dr. Ball, a similar course (these courses are limited to five men). Professor Borchardt, a course on surgical diagnosis. Dr. Helbing, a course on orthopædic surgery on selected patients. Dr. Zondels, a course on surgical therapy. Dr. Wolff, operative surgery on the dog and on the cadaver, 4 weeks, thrice weekly two hours, fee 60 marks. Professor Schmieden and Dr. Joseph, special courses on hyperæmia, fee 25 marks.

*Gynæcology.*—Professor Nagel, 4 weeks, 60 marks. Dr. Landau, diagnosis, treatment, and operations. Dr. Runge and Dr. Blumreich, special courses in gynæcology, with practical work on the living and on the phantom.

*Midwifery.*—Dr. Martin, a special course at the Charité, daily. Dr. Bosslar, a similar course. In both cases membership is limited to five and the fee is 300 marks.

*Skin.*—Dr. Joseph, Dr. Saalfeld, Dr. Frank, Dr. Blasko (all English speaking) give courses by arrangement.

*Nose, Throat, and Ear.*—Dr. Halle, a special course, 75 marks, with operative work on selected cases, 100 marks. Dr. Meyer, practical laryngo and rhinosopy, 60 marks. Similar courses by five other specialists.

*Eye.*—Four special courses, all by English-speaking specialists.

*Neurology.*—Professor Oppenheim, a monthly course, fee 40 marks. Drs. Ziehen, Lewandowsky, Jacobson, Cohn, and Flatau give similar courses.

*Pediatrics.*—Five special courses.

*Cystoscopy.*—Five special courses, with opportunities in each for practical work on normal and pathological bladders, fees from 60 marks.

*Pathology.*—Four special courses, all practical, fees from 40 marks.

*Bacteriology.*—Special courses at various fees. A knowledge of German is absolutely necessary.

*Blood.*—Four special courses. Anatomy, histology, and embryology, special courses with dissections and microscopical work, from one to three months, at fees averaging 100 marks.

*Dietetic Cooking.*—A special course lasting 4 weeks, fee 30 marks.

*Röngten-ray Work.*—A special course lasting a month.

In addition, special courses can usually be arranged. Thus, as there are several men here at present who wish to become proficient in spinal anæsthesia, the Association has arranged a special course, with opportunities for operative work, at the low fee of 60 marks for 6 weeks' work, thrice weekly, the number of men in the course being limited to five. It must be borne in mind that all these courses are given by specialists at recognised poly and internal clinics—such as the Charité, the Royal, the Ear or Eye, Virchow, and at the University laboratories. There can thus be no comparison with these courses and the coaching classes that may be attended in London or Edinburgh, where the instructors are usually junior men who have not had over much experience, and who have far less clinical material to work with. The opportunities for operative work, for example, are such as can never be obtained in London under existing conditions.

We have dealt at some length with the Anglo-American Association because it is a body that is doing very good work and which appears to be but little known outside Berlin. Its objects are praiseworthy, and if it can bring the merits of Berlin as an educative centre for post-graduate students more prominently before British practitioners, it will do very excellent work. Most British practitioners who go to the Continent for study go to Vienna, simply because they do not know of the great opportunities that are available at Berlin. Study at other German centres, such as Heidelberg and Munich, is by no means so easy as here at Berlin, where the clinical material is overwhelmingly superior.

Besides the Anglo-American, there are in Berlin several other post-graduate associations arranging special courses for practitioners. These are, of course, without exception, German courses, and no one who intends to join them should do so unless he is fairly proficient in the language. As a general rule these courses are cheaper than those given to foreign students, but they are open to the latter just as much as to German practitioners. The best are given during the vacations, that is, during the months of March and November. For example, at present Professor Pels Lensden, of the Charité Polyclinic, is giving a course on operative surgery, two hours daily, for a month, the operations, which are specially designed for familiarising the student with intestinal work, being done on the dead body and on dogs. Thrice weekly he gives his class a two-hours' demonstration on clinical surgery, with actual operative work (usually under local anæsthesia) on the living subject. Such a course is, of course, out of the question in London, but here it is by no means unusual.

Among the larger and more important associations, the following may be mentioned : Central Committee for Post-graduate Study in Prussia (Office Kaiserin Friedrichhause, Luizen-Platz 6, N.W.), which issues a long list of special courses at very much reduced fees or, in the majority of cases, entirely gratuitous. To the gratuitous courses, however, foreign students are not admitted except by courtesy of the demonstrating professor. To courses for which a small honorarium is payable, foreign students are admitted on the same terms as German graduates, provided they make early application and there is room for another member. A vacation course is, however, usually crowded, especially as the membership is limited to ten or less. The Docentenverein für Ferein-Kurse (Graduate Association for Vacation Courses) (Office H. Melzer, Langenbeck-hause Zieglestrasse 10-11) The Verein für ärztliche Fortbildung (Association for Post-graduate Study) (Office Buchhandlung Otto Enslin, Karlstrasse 32), the Verein für Aerzte-Kurse (Association for Graduate Courses) (Office Medizin, Warrenhaus, Karlstrasse 31), and finally the Vereinigung zur Veranstaltung von Kursen für practische Aerzte (Society for the Establishment of Post-graduate Courses) at the Buchhandlung Otto Rothacker, Friedrich-strasse 105b, all issue lists of special courses very similar to the first-mentioned association. A note to any of the secretaries is usually sufficient to elicit all requisite information as to hours, membership, fees, and requirements generally.

